# Adolescents’ agency toward climate change: development and validation of scales for individual, proxy, and collective modes

**DOI:** 10.3389/fpsyg.2025.1532409

**Published:** 2025-05-05

**Authors:** Jennifer Cunha, Juliana Martins, José Carlos Núñez, Guillermo Vallejo, Pedro Rosário

**Affiliations:** ^1^Psychology Research Center, School of Psychology, University of Minho, Braga, Portugal; ^2^Departamento de Psicología, Universidad de Oviedo, Oviedo, Spain

**Keywords:** human agency, climate change, climate action, adolescents, scale validity, measurement invariance

## Abstract

In a rapidly evolving world, human agency serves as a driving force to shape a more sustainable future. The climate crisis is an example of how individuals must be proactive and take action to mitigate this environmental problem through three modes of agency advocated by Bandura: individual, proxy, and collective. This is even more relevant for adolescents, who will most suffer climate change consequences. However, instruments assessing adolescents’ agency modes toward climate change are still lacking. To address this gap, we present the development and validation of three theoretically based scales for assessing each mode of adolescents’ agency toward climate change (AGENTC2). The AGENTC2-Scales were developed based on a literature search, expert review, and consultation with a panel of adolescents. The AGENTC2-Scales were then empirically tested with 1,114 adolescents, and their psychometric properties were assessed, providing evidence of validity (i.e., content, structural, and convergent), measurement invariance (sex and school grade), internal consistency, and test–retest reliability. Data showed that the AGENTC2-Scales can be used to measure each mode of agency toward climate change and their properties validly and reliably. Further research is needed to extend the validation of the scales in other countries.

## 1 Introduction

Among the problems that threaten sustainable living on our planet, climate change (CC) requires rapid action (United Nations, 2015; Waddock, 2013). Everyone must be called to take action to mitigate CC. Not disregarding the undeniable role of macro and political actions to mitigate CC, young people are relevant players in this process as they are more susceptible to the CC immediate and lifelong effects than adults (Bandura and Cherry, 2020; Sanson et al., 2019). In this context, there is a call to consider “young people as active agents and protagonists for change” (Sanson et al., 2019, p. 203). The construct of human agency by Bandura (2006b) fits this purpose. In this view, individuals can influence the course of their lives by acting in various modes; i.e., individually, collectively, or by influencing others (Bandura, 2006b). This agentic approach to action allows young people to extend the reach of their actions toward mitigating CC beyond their individual spheres of functioning. However, based on the literature review, no instrument purposefully developed to assess adolescents’ agency toward CC was found. Therefore, the present study intends to develop and validate new scales theoretically grounded on Bandura’s proposal for assessing adolescents’ agency modes toward CC.

### 1.1 Conceptualizing and connecting human agency with CC

Social cognitive theory follows an agentic perspective toward human psychosocial functioning, highlighting the human agency fundamental role in individuals’ self-development, adaptation, and change over time (Bandura, 2001). Human agency is defined as the human capability to intentionally influence one’s functioning and the course of life through one’s actions (Bandura, 2006b).

According to Bandura (2006b), this construct includes four core properties – intentionality, forethought, self-reactiveness, and self-reflectiveness. Nevertheless, in 2018, Bandura embedded the intentionality property in the agency construct (e.g., stating that “to be an agent is to intentionally produce certain effects by one’s actions”, p. 130) and described the human agency as subsuming three core properties (i.e., forethought, self-reactiveness, and self-reflectiveness). We followed Bandura’s (2018) understanding of human agency in this work. In forethought, individuals extend their agency in time, setting goals, designing action plans consistent with their goals, and anticipating the potentially achievable outcomes to foster their motivation (Bandura, 2006b, 2018). For example, individuals can proactively take steps to mitigate CC by anticipating the potential long-term consequences of their actions on the environment and future generations. In self-reactiveness, individuals activate their self-regulation processes (e.g., monitoring their CC mitigation behaviors and efforts, making adjustments in their plans while performing it, attributing themselves rewards or sanctions) to execute the pre-established action plans and ensure control over their behavior against their standards (Bandura, 2001, 2018). Finally, in self-reflectiveness, individuals reflect upon their behavior and evaluate their functioning (i.e., personal efficacy, values, and the meaning of their purposes, for example, CC mitigation). Through this metacognitive exercise, individuals examine their thoughts and actions and consider possible corrective adjustments (e.g., alternative strategies and pro-environmental behaviors) favoring the reach of their pursuits (Bandura, 2006b). In sum, according to Bandura’s theory of human agency, individuals are characterized as being simultaneously forethinkers, self-regulators, and self-examiners, which allows them to adopt an agentic approach in their lives (Bandura, 2018).

Moreover, these three core properties can be displayed through three modes of agency – individual, proxy, and collective (Bandura, 2006b, 2018). The individual mode includes actions and activities over which individuals exercise direct control (e.g., daily individual CC mitigation behaviors such as reducing consumption, unplugging electronic devices not in use, using public transportation, and adopting a vegetarian diet; Koskela and Paloniemi, 2023). However, actions addressing a complex environmental problem, such as CC relying exclusively on the individuals’ control, are limited. For example, individuals do not directly write the laws of the country or set the policies and practices followed in institutional contexts, such as in their workplaces or, in the case of adolescents, in their schools or home, where adults usually lead decisions.

All considered, contextual factors may favor or hinder individual agency. For example, using public instead of private transportation is only possible if these are available in the community; also, adopting a vegetarian diet may be difficult to sustain if vegetarian options are not part of the menu of restaurants, stores, and local markets (Koskela and Paloniemi, 2023). To overcome these contextual barriers, individuals can exercise their socially mediated proxy agency by “influencing others who have the resources, knowledge, and means to act on their behalf to obtain the outcomes they desire” (Bandura, 2018, p.131). In other words, individuals can act through better-equipped or positioned individuals to propose solutions favoring pro-environmental behaviors (e.g., reaching environmental institutions or politicians whose actions directly impact society). Furthermore, specific goals and purposes can only be achieved through group effort (e.g., activists’ actions to draw attention to the triggers of CC, which are rooted in human activity such as industrialized activities and low-efficient energy use, responsible for high emissions of greenhouse gasses). When working together with others and sharing common goals, individuals are likely to follow a multiagent model of agency termed collective agency (Bandura, 2018). In sum, while acting intentionally, individuals can exercise their agency: (i) on their own, (ii) through influencing people with knowledge and resources to act on their behalf when it is not in their power to do something, and (iii) merging their efforts with group efforts to achieve common major goals. This way, people could follow an ecological approach, increasing the impact of their direct and indirect actions on diverse situations as CC mitigation.

### 1.2 Literature review: instruments assessing agency

The current study followed the literature recommendations to develop scales assessing agency (e.g., Boateng et al., 2018; Tsang et al., 2017; Yasir, 2016). Concretely, we followed two steps before building our instruments: (i) an analysis of the literature on the instruments addressing human agency and (ii) instruments on human agency in the domain of CC.

Firstly, we conducted a thorough literature review on human agency. Searches using several combinations of the terms “agency,” “human agency” and “agentic” AND “instrument,” “questionnaire,” “survey,” and “scale” were run on Scopus, Web of Science (all databases), ERIC, and Google Scholar databases. As a relevant finding of these systematic searches, we highlight the recent literature review of Cavazzoni et al. (2022), addressing the quantitative instruments used to measure children, youth, and adults’ agency across distinct contexts. The detailed analysis of the 34 included studies revealed that researchers grounded their studies on diverse theoretical frameworks for agency, which translated into the adoption of distinct definitions and instruments.

Despite distinct and focusing context-related specificities, the definitions of human agency cited in this pool of papers seem to share core characteristics, such as intentionality, choice, and control over actions (e.g., Bryan et al., 2014; Zimmerman et al., 2019). Moreover, most of the instruments used in the sampled studies were built to assess diverse forms of agency (e.g., critical, political, sexual, and moral agency). Still, instruments built originally to assess other constructs were also used to measure agency within the targeted populations (e.g., Snyder’s Children Hope Scale, Poteat et al., 2018; Adolescent Autonomy Questionnaire, Beyers et al., 2003; Racial Cohesion Questionnaire targeting adults, Bentley-Edwards, 2014). The analysis of the retrieved studies also revealed that, in some studies, other constructs were added to the realm of the agency construct, such as self-efficacy (Alkire, 2005; Hitlin and Elder, 2006), autonomy (Beyers et al., 2003), and empowerment (e.g., Alkire, 2005; Berhane et al., 2019; Pick et al., 2007; Zimmerman et al., 2019). Moreover, it was also possible to identify different operationalizations of the agency construct, for example, as a single variable (e.g., Lautamo et al., 2020; Steckermeier, 2019) or as a composite variable comprising varied dimensions (e.g., dispositional, motivational, and positional, see Vaughn et al., 2020b), domains (e.g., voice, behavioral control, decision making, see Zimmerman et al., 2019) or indicators (e.g., perceived control, sense of self-efficacy, work ethic, see Burger and Walk, 2016).

The overall results of our database searches revealed a similar trend to that reported in Cavazzoni et al.’s (2022) review. Research on agency has extended its reach to cover several populations (i.e., children, youth, and adults), contexts (e.g., educational, moral, and political), and domains (e.g., career development, e.g., Betz and Hackett, 1987; Yoon, 2011, 2019; exercise and physical activity, e.g., Blacksher and Lovasi, 2012; Shields and Brawley, 2006; education, e.g., Jääskelä et al., 2016; Luo et al., 2019; Stenalt and Lassesen, 2022; Vaughn et al., 2020a; environment, e.g., Kachel, 2012; Oliveira et al., 2015). This extended coverage of the agency construct may help explain the high variability found in the definitions and instruments used.

Interestingly, just four studies in our pool were framed on Bandura’s (2006b, 2018) Human Agency Theory: an unpublished doctoral dissertation (Yoon, 2011), one empirical paper (Code, 2020), and two theoretical papers (Koskela and Paloniemi, 2023; Yoon, 2019). For the purposes of the current research, we describe briefly the two empirical studies and discuss the contributions of the two theoretical papers for the CC domain. Yoon (2011) developed the “Assessment of Human Agency” (AHA) instrument. The AHA was purposefully designed to assess the agency of adults (i.e., employees and traditional and non-traditional university students), addressing aspects related to individual performance in an organizational context. Centered on the career domain, this instrument allows mapping individuals’ strengths and deficiencies among the four core properties of individual agency mode (Bandura, 2006b).

Code (2020) developed the Agency for Learning Questionnaire (AFLQ) to assess multidimensional aspects of undergraduate students’ agency in learning. This author grouped the items (extracted from other instruments) according to the four agency core properties (Bandura, 2001, 2006b): intentionality, forethought, self-reactiveness [self-regulation], and self-reflectiveness [self-efficacy] (see Code, 2020, pp. 2–3). Notwithstanding, the selected items do not represent all aspects included in Bandura’s definitions of the core properties. Both instruments (i.e., AHA instrument, Yoon, 2011, and AFLQ, Code, 2020) are exclusively centered on the individual agency mode.

The two theoretical papers focused on exploring and providing relevant inputs on human resource development (Yoon, 2019) and conceptualizing human agency for sustainability transformations (Koskela and Paloniemi, 2023). For example, the article of Koskela and Paloniemi (2023) contributes to bridging Bandura’s theory with the CC domain, which was particularly relevant for our purposes.

Secondly, we narrowed our focus while searching for literature addressing instruments on human agency in the domain of CC or environment. The searches combined “climate change,” “human agency” and “measures,” “scales” or “instruments.” The sample of studies found was not grounded on Bandura’s theoretical framework and followed a qualitative approach (e.g., Blanchet-Cohen, 2008; Muller and Wood, 2021; Oliveira et al., 2015; Toivonen, 2022; Trott, 2020). In sum, based on the results of the searches performed, no instrument theoretically grounded in Bandura’s theory has been developed to assess adolescents’ three modes of agency toward CC.

### 1.3 Why studying adolescents’ agency toward CC?

Adolescence is a pivotal developmental phase marked by the development of the capacity to think beyond concrete phenomena – abstract thinking (e.g., Byrnes, 2006), and an emerging connection and interest in global society problems (as CC) in a more independent way (Holden, 2007). Throughout this developmental period, parallel to the development of diverse skills (e.g., emotional intelligence, critical thinking, socialization, self-regulation; Zimmer-Gembeck and Collins, 2006), adolescents acquire a growing understanding of the causes and consequences of broader sustainability and environmental issues while gradually experiencing autonomy in decision-making and behavior (Ojala and Lakew, 2017). The capacity to think abstractly and critically about certain phenomena allows them to make choices that fit their understanding of their world and their role as citizens (Zimmer-Gembeck and Collins, 2006). As citizens of today and of the future, adolescents can either exacerbate CC through unsustainable lifestyles or contribute to its solution by adopting climate-friendly habits, influencing their parents and peers, and advocating for change through political protest (O’Brien et al., 2018; Metzger et al., 2010). However, despite facing the same developmental changes, not all adolescents are in the same stage of development (e.g., middle and high school students), and neither exhibit the same level of knowledge, concern, and willingness to act in similar areas as in the case of CC. Literature indicates that although adolescents’ CC knowledge tends to increase with age, their willingness to act on this problem tends to decrease (Lee et al., 2020). On the other hand, some authors (e.g., Olsson and Gericke, 2016; Otto et al., 2019) describe a fluctuation in environmental concern across adolescence that tends to be reestablished during emerging adulthood, called “adolescence dip.” For these reasons, adolescence could be a critical period since it could influence climate engagement in adulthood (Nash et al., 2020).

### 1.4 Study purpose and hypotheses

To address CC, it is necessary to mitigate its triggers rooted in human activity responsible for high greenhouse gas emissions (Swim et al., 2011). As Sanson et al. (2019) alert, this is even more relevant for adolescents, given that younger generations will suffer the most consequences of CC. However, not all adolescents are equally interested and committed to combat CC. Importantly, despite the increasing development of several skills throughout adolescence (e.g., Byrnes, 2006; Zimmer-Gembeck and Collins, 2006), empirical data also indicate that this period can also be marked by a decrease in CC action (e.g., Lee et al., 2020; Otto et al., 2019). These data from developmental and environmental psychology literature underscores the need to study adolescents in different stages of development, in the case of the current study, early and middle adolescence, that approximately correspond to middle and high school levels.

Bandura’s theory of human agency provides insights into the CC problem by emphasizing the role of the individual, proxy, and collective agency in initiating and sustaining action and behavior change (Bandura, 2018). These agency modes are particularly relevant when dealing with CC, where solutions must be built at individual and collective levels and by influencing people with knowledge, resources, or decisive power (Bandura and Cherry, 2020; Ojala, 2012; Sanson et al., 2019).

However, to the best of our knowledge, literature lacks an instrument (i) assessing the three agency modes proposed by Bandura, (ii) targeting adolescents, or (iii) focusing on the CC mitigation domain. To fill this three-fold gap, the current research aims to develop and validate new theoretically based scales for the Portuguese population, assessing adolescents’ individual, proxy, and collective modes of agency toward CC. Following research guidelines for developing and validating instruments (e.g., Boateng et al., 2018; Tsang et al., 2017), we conducted the current study in two phases: (i) development process of the AGENcy Toward Climate Change Scales (AGENTC2-S), and (ii) AGENTC2-Scales testing and validation.

**Figure 1:**
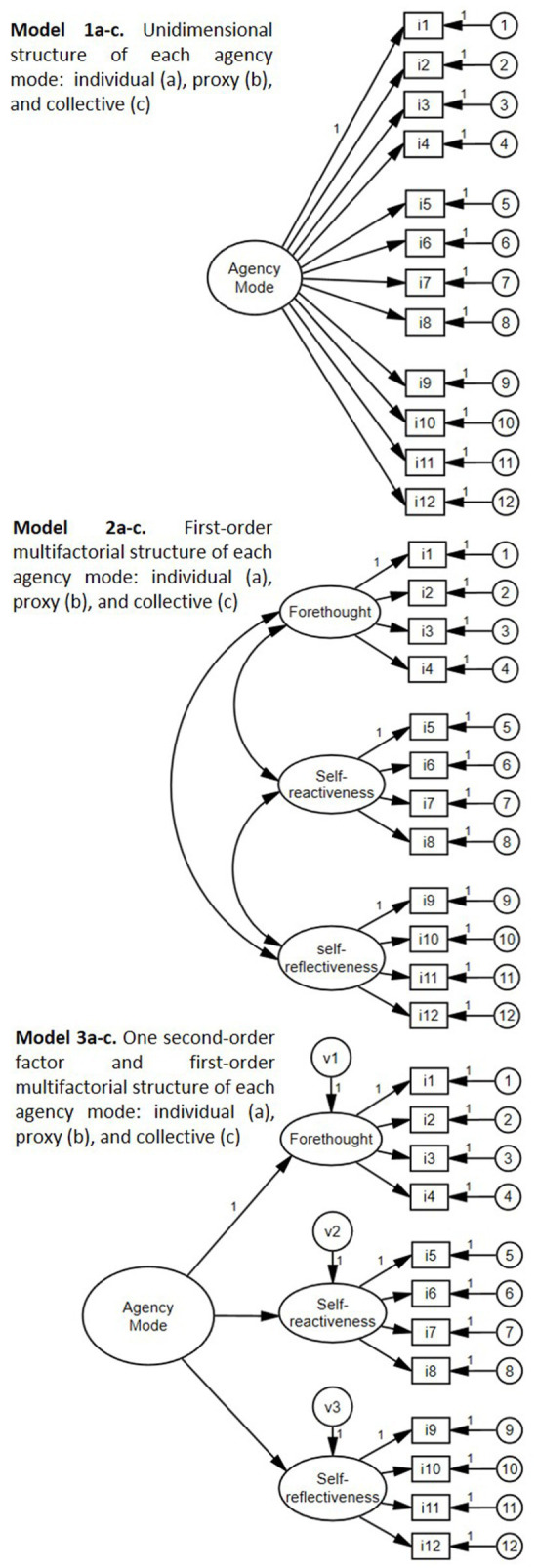
Hypothetical models for testing the structural validity of each agency mode scale.

These new scales for assessing the three agency modes are grounded in the human agency theory by Bandura (2018); therefore, we hypothesized that each scale has a multidimensional and multilevel structure (H1). Specifically, the first level encompasses three factors, i.e., properties (i.e., forethought, self-reactiveness, and self-reflectiveness), loading on a second-order factorial level, the overall agency mode (i.e., individual, proxy, or collective, see Figure 1 – Model 3a-c). Secondly, we hypothesized the invariance of this model across sex (H2) and grade level (H3), i.e., the model measures the same construct for girls and boys, as well as for adolescents in different grade levels (7th–12th).

Concerning reliability, we hypothesized that individual (H4), proxy (H5), and collective (H6) agency mode scales show high internal consistency at the first and second waves of data collection (Cronbach’s alpha ≥ 0.70). We also hypothesized high correlations between both data collection waves for each agency mode scale, indicating test–retest reliability (H7–H9).

To confirm convergent validity, we tested whether agency modes were positively and statistically correlated to theoretically related variables. For example, prior research has shown positive relationships between pro-environmental or CC mitigation behaviors and CC self-efficacy (e.g., Busch et al., 2019), nature connectedness (Krettenauer et al., 2020), and CC concern (e.g., Lawson et al., 2019). Hence, we hypothesized that adolescents’ agency modes toward CC are positively related to CC mitigation behaviors (H10), self-efficacy to combat CC (H11), nature connectedness (H12), and CC concern (H13).

Lastly, literature on learning agency, self-regulation, and pro-environmental or CC mitigation behaviors shows similar results. Girls tend to report more agentic and self-regulation learning behaviors (e.g., Martins et al., 2024; Motie et al., 2012; Zimmerman and Martinez-Pons, 1990) and pro-environmental or CC mitigation behaviors (e.g., Fielding and Head, 2012; Zeeshan et al., 2021) than boys. In addition, throughout adolescence, pro-environmental or CC mitigation behaviors tend to decline (e.g., Krettenauer, 2017; Negev et al., 2008). All considered, we hypothesized that girls report higher agency toward CC than boys (H14), and agency scores decline throughout schooling (H15).

## 2 Materials and methods

This study was conducted under the project “There’s no planet B” (PTDC/PSI-GER/1892/2021). Ethical approval for the project studies was obtained from the Ethics Committee for Research in Social and Human Sciences (CEICSH 112/2022) and the Data Protection Officer of the University of Minho. The Portuguese Ministry of Education also provided permission to collect data in schools nationwide.

### 2.1 Phase 1: Development process of the AGENTC2-S

To build questionnaires to assess adolescents’ modes of agency toward CC, first, we developed an item pool based on the AHA instrument (Yoon, 2011) that is grounded on Bandura’s conceptualization of agency. These efforts were preceded by an extensive literature review on the human agency construct (see section “Literature review: Instruments assessing agency”). In the following step, to improve item formulation and calculate content validity, we asked experts on human agency to evaluate the quality of a pool of items (i.e., the AGENTC2-S items) selected to measure the three modes of agency. Finally, we interviewed adolescents from middle and high school levels to learn their understanding of the AGENTC2-S items. All these processes are detailed below.

#### 2.1.1 Questionnaire construction: item generation, formulation, and response format

Following a deductive approach, we used Bandura’s (2018) theory to conceptualize agency, its three core properties, and modes. Next, we examined the literature on the CC topic to define the construct and understand how it could be associated with the agency construct and assessed as the principal domain. In the following step, we examined the existing instruments assessing agency. As previously mentioned, we found two instruments assessing human agency grounded on Bandura’s theory (i.e., AHA and AFQL). These instruments were developed to map adults’ agency in relation to their professional careers (i.e., AHA; Yoon, 2011) and learning (i.e., AFQL; Code, 2020). Importantly, these instruments approached human agency as a result of individual efforts exclusively. For these reasons, these instruments do not fit our purposes. However, after analyzing the items of both instruments regarding representativeness and fit with Bandura’s conceptualization of the three core properties, we selected the AHA instrument as a starting point for developing the AGENTC2-Scales. Preliminary AGENTC2-S items for the individual agency mode were built upon the AHA initial version and adapted to the CC domain. The content and fitness of each item were then analyzed against Bandura’s definition of each agency property. The items for the proxy and collective modes of agency were built from those of the individual mode. This procedure allowed us to maintain the content representative of each core property and just change the item formulation to match proxy and collective modes. It is important to note that the AHA items were formulated in English. However, considering that our target population is Portuguese adolescents, the new items of the AGENTC2-S for each mode were formulated in Portuguese language and adapted to their comprehension level.

During this stage, while formulating the items, efforts were made to keep them simple, straightforward, and written in language familiar to the intended respondents, as recommended by literature (Boateng et al., 2018; Tsang et al., 2017). Moreover, acknowledging that adolescents are usually not interested in completing long questionnaires with complex items, whenever possible, details considered unnecessary (e.g., concrete examples of individual, proxy, or collective actions toward CC) were avoided or removed. Further, following Krosnick and Presser’s (2009) recommendation, we opted to provide respondents with a Likert-type response scale with five points to ensure sufficient variance among the intended respondents. Moreover, we decided to use frequency descriptions (i.e., 1 = never to 5 = always) to facilitate adolescents’ identification and quantification of their self-perceived thoughts and behaviors.

Following Schinka et al.’s (2012) recommendation that the initial pool of items should be at least twice as long as the desired instrument to provide a necessary margin to select an optimal combination of items, the first version of the AGENTC2-Scales comprised 69 items in total (23 items per each mode). However, in the subsequent revision of the items, we eliminated 18 due to their overlap with other items. Therefore, the preliminary version of the AGENTC2-S comprised 51 items symmetrically distributed among the three agency modes (i.e., 17 items per mode). Moreover, within each mode, items were distributed according to the three core properties of agency (i.e., forethought [5 items], self-reactiveness [5 items], and self-reflectiveness [7 items]). To maintain representativeness and consistency across agency properties and modes, the research team’s decisions regarding including or excluding new items in one mode were extended to the other modes. This procedure was applied in all phases of the instrument development (e.g., while accommodating the inputs provided during the evaluation performed by experts and target population judges).

#### 2.1.2 Evaluation by expert judges

Researchers with experience in instrument development and validation recommend recruiting 3–10 experts on the topic as independent judges (e.g., Lynn, 1986; Polit et al., 2007). In this study, we invited five researchers with expertise in human agency and instrument development to review the AGENTC2-Scales independently. Hence, a detailed package with information and materials (see Elangovan and Sundaravel, 2021; Yusoff, 2019) was sent to the five expert judges via email. This package included (i) an invitation letter, (ii) a description of the purpose of the AGENTC2-Scales, (iii) a literature review on the human agency construct, (iv) an explanation of the expectations for the expert judges’ work, (v) the full version of the AGENTC2-Scales paired with the fulfillment instructions, types of action explanations (i.e., individual, proxy, and collective) and respective examples, as intended to be presented to adolescents, (vi) the instructions to evaluate the items (e.g., check whether items wording are simple, accurate or the content can be perceived as offensive or biased by respondents) and (vii) the instructions for assessing content validity (together with an online content validation form to calculate the Content Validity Index, CVI).

In sum, the expert judges were requested to critically review all the items and identify (mis)matches with the definitions of the corresponding modes and properties of agency on the CC domain. This process resulted in minor changes, such as rewording or clarifying a few items. For example, the verb “I anticipate possible consequences…” was signaled by some experts as potentially difficult to understand by adolescents, so, as suggested, we changed the item to “I think about possible consequences….” Also, in some items where examples of actions were provided, experts suggested removing them to avoid confusion or biased answers. Finally, the experts rated all items regarding their (i) relevance, (ii) clarity, (iii) simplicity, and (iv) ambiguity on a four-point scale (e.g., 1 – “Irrelevant” to 4 – “Very relevant”) considering the property and mode of agency the items were supposed to assess. This allowed us to calculate the CVI of the AGENTC2-S.

Overall, the instrument showed an appropriate level of content validity. The item-level CVI of the three agency modes ranged between 0.80 and 1.00, and the scale-level CVI of the three agency modes ranged between 0.92 and 1.00, which indicates excellent content validity scores (see Lynn, 1986; Polit and Beck, 2006; Polit et al., 2007). The CVI of the instrument using the universal agreement approach ranged between 0.71 and 1.00 for each agency mode. These data indicate that the adolescents’ agency modes toward CC seem to be comprehensively sampled by the items of the AGENTC2-S.

#### 2.1.3 Evaluation by target population judges

After the expert assessment, the research team contacted through email five schools near the University to recruit students for a cognitive interview (Boateng et al., 2018). The school principals resent the invitation (containing the informed consent attached) to the students’ parents or guardians. Ten parents or guardians responded positively, however one student did not provide assent. Therefore, the preliminary version of the AGENTC2-S was administered to nine adolescents (ages from 12 to 17 years old) in person or through a Zoom meeting. In this individual meeting, which consisted of a spoken reflection, items were presented in two ways: (i) organized per mode (i.e., first, all items of the individual mode followed by the items of the proxy mode; and then the items of the collective mode) and (ii) organized in a table comprising the items of the three modes numbered and presented in three columns in parallel (see Supplementary Table S1). Adolescents were asked to select the presentation approach more likely to favor the fulfillment of the scales and justify their options. All adolescents selected the latter presentation approach. The main reasons were twofold: the visual aspect and the lower effort required to fulfill the equivalent items for three modes in the same row. Moreover, the nine adolescents were asked to think aloud while completing the questionnaire and explain their understanding of the individual items. While reading the items, adolescents were asked to identify words that they did not know or comprehend and explain in their own words their understanding of items or of specific words previously identified as potentially problematic by the research team (e.g., “strategies,” “plan” “collective actions”). The feedback retrieved from this pilot led to the exclusion of overlapping items or of those that, due to their theoretical similarity, triggered similar responses across respondents (i.e., low variability). Overall, 15 items (i.e., five per agency mode) were removed from the pool of the AGENTC2-S items.

The final version of the AGENTC2-S comprised 36 items symmetrically distributed within the three agency modes and core properties (i.e., 12 items per agency mode, each with four items for every property). To avoid the participants’ perception of item repetition, the items were alternated (i.e., item 1 of forethought was followed by the first item of the self-reactiveness and self-reflectiveness properties, respectively; see Supplementary Table S2).

### 2.2 Phase 2: AGENTC2-scales testing and validation

After concluding the developmental process, we tested and validated the AGENTC2-S. Specifically, as we aimed to test a clear, theoretically driven factor structure of the construct of human agency applied to the CC domain, we assessed the scale’s factor structure using a confirmatory factor analysis (CFA). We also assessed the measurement invariance, reliability, and convergent validity of the scales.

#### 2.2.1 Participants and procedure

The guidelines for estimating the sample size needed for instrument validation vary (Tsang et al., 2017). Overall, 300 respondents are considered good-, 500 are very good-, and more than 1,000 are an excellent sample size (Comfrey and Lee, 1992). To recruit the participating adolescents, the principal investigator sent an invitation email to schools nationwide. After acceptance, in accordance with the Declaration of Helsinki, written informed consent (i.e., study goals, procedure) was provided to the parents or guardians of the participating students. All participants were informed about their voluntary participation and data confidentiality.

Participants were 1,114 adolescents from the middle (*n* = 722) and high school (*n* = 392) levels, aged between 11 and 19 (*M* = 12.98; SD = 1.01) and between 13 and 19, (*M* = 15.83; SD = 0.88) respectively. Among middle schoolers (50.0% female), 266 were enrolled in the 7th grade, 210 in the 8th grade, and 246 in the 9th grade. Among high schoolers (59.4% female), 119 were enrolled in the 10th grade, 200 in the 11th grade, and 73 in the 12th grade. Participants were students of 13 schools from distinct parts of the country (i.e., nine schools from the North, two from the Center, and two from the South). All respondents reported that CC was addressed at least in one school subject at some point in their academic path, and 2.8% reported having already participated in a school climate strike or protest. Most participants (91.0%) reported Portugal as their place of birth.

Data were collected in two waves with a three-week interval (i.e., T1 and T2, between October and December 2023). The questionnaire was delivered to participants in person during the student’s school schedule. The questionnaire comprised the AGENTC2-Scales and additional scales to examine convergent validity. To prevent overloading the participants, at T1, one group filled out version A (i.e., self-efficacy), and another filled out version B (i.e., nature connectedness and CC concern). At T2, all participants filled out the same scale (i.e., CC mitigation behaviors).

To gather information concerning students’ understanding of the agency modes items, at T1, students were provided with an “I do not understand” option on every item of the questionnaire. Following Wang et al. (2014), if more than 1% of the participants chose this option in the same item, the item would be revised for the T2. No participants selected this option, corroborating the feedback provided by the target population judges.

#### 2.2.2 Measures

The participants were asked about sociodemographic data (e.g., sex, age, place of birth), the school subjects addressing climate change, and their participation in school climate strikes or protests. Then, multi-item scales, briefly described hereafter, were delivered to participants.

##### 2.2.2.1 AGENcy Toward Climate Change Scales (AGENTC2-S)

Three 12-item scales were developed to assess adolescents’ individual, socially mediated by proxy, and collective agency toward CC. In total, participants responded to 36 agency items (see English and Portuguese Versions, Supplementary Tables S2, S3) on a five-point Likert scale ranging from “never” (1) to “always” (5). Psychometric properties are provided in the results section.

##### 2.2.2.2 CC mitigation behaviors

According to the United Nations Environment Programme [UNEP] (2020, 2022), behaviors to mitigate CC should address: (i) energy, (ii) agriculture, food, and waste, (iii) building and cities, (iv) nature-based solutions, (v) industry (e.g., reduce, reuse, repair and recycle), and (vi) transport sectors. Grounding on the literature review and the United Nations Environment Programme [UNEP] (2020, 2022), we build a 13-item scale targeting CC mitigation behaviors in the sectors adolescents may have more control and autonomy. Sectors selected were as follows: energy (e.g., “I turn off the lights when I leave a room”), agriculture, food, and waste (e.g., “I choose to do more vegetarian meals”), industry (e.g., “I recycle paper/cardboard, plastics/metal, and glass wherever I am”), and transport (e.g., “I walk or cycle instead of using a car”). The items were scored on a five-point Likert scale ranging from “never” (1) to “always” (5). In this study, Cronbach’s alpha was 0.74.

##### 2.2.2.3 Self-efficacy to combat CC

Adolescents’ self-efficacy beliefs to combat CC was assessed through a nine-item scale querying their confidence level to mitigate CC. According to Bandura (1989), p. 1175, self-efficacy refers to “people’s beliefs about their capabilities to exercise control over events that affect their lives”. Grounding on this definition of self-efficacy, and following Bandura’s guide to constructing self-efficacy scales (Bandura, 2006a), we selected and adapted nine items from instruments used to assess self-efficacy beliefs in environmental and CC domains (i.e., Kolenatý et al., 2022; Moser and Seebauer, 2022; Muroi and Bertone, 2019; Sarrasin et al., 2022). Moreover, the response scale was adapted to allow adolescents to rate their confidence level to mitigate CC and minimize response bias. Hence, each item begins with the phrase “How confident are you that you can …” and is completed with statements such as “… do something to help reduce climate change” or “…suggest how your school could help reduce climate change” or “combat climate change together with your family, school and community.” Adolescents answered on a five-point Likert scale ranging from “not confident at all” (1) to “Very confident” (5). Cronbach’s alpha was 0.91 (T1 and T2).

##### 2.2.2.4 Nature connectedness

To assess adolescents’ connectedness with the natural environment, we adapted the Schultz (2002) scale, Inclusion with Nature in Self. This is a visual measure comprised of seven pairs of overlapping circles (i.e., “Self” and “Nature”) with growing levels of intersection. For the purpose of this study, we highlighted the intersection area of the circles (a familiar concept to adolescents from mathematics classes) to facilitate adolescents’ comprehension of the response options. The larger the intersection area, the higher the nature connectedness. Participants were asked to select the pair of overlapping circles representing the level of intersection that best describes their connectedness with nature.

##### 2.2.2.5 CC concern

To assess adolescents’ CC concern, we used the item “How worried are you about climate change?” from the work of Lawson et al. (2019). To provide adolescents with a graphical perspective regarding the CC concern, response options, ranging from “not at all worried” (1) to “very worried” (5), were embedded in a thermometer image (see Supplementary Figure S1).

#### 2.2.3 Data analysis

The data were analyzed in several stages. Initially, we examined the descriptive statistics and the correlation matrix. A few missing values (3.18%) were found, and the maximum likelihood procedure was followed to complete the information.

As the developed scales are theoretically driven, CFA is the most appropriate and robust analysis to test the hypothesized structure (see Lambert and Newman, 2023) of human agency modes and properties proposed by Bandura (2018). The analyses were conducted using the AMOS software to study our first two goals (multidimensional and multilevel structure and invariance across sex and school level). For each of the three agency modes, three models were fit. A total of nine models examined the latent structure of the three scales. For each of the three agency modes, three models were fit as follows: a single-factor model (a single general factor explained the 12 items) – model 1- (see Figure 1 – Model 1a–c), a model of three first-order factors -model 2- (see Figure 1 – Model 2a–c) (the three agency proprieties: forethought, self-reactiveness, and self-reflectiveness), and a two-level factorial model -model 3- (see Figure 1 – Model 3a–c) (the three-factor model [proprieties] plus a general factor at the second factorial level [agency mode]).

Confirmatory factor analysis results were evaluated with the following model fit indexes: Chi-square, AGFI, TLI, CFI, SRMR, and RMSEA. There is evidence of a good fit when *χ*^2^ has *p* > 0.05, AGFI and TLI ≥ 0.90, CFI ≥ 0.95, RMR and RMSEA ≤ 0.06. The selection of the best structural model is made based on the AIC and BIC statistics (the best-fit model is that with the lower AIC and BIC values). According to Cheung and Rensvold (2002), data show invariance across sex and school grade when ΔCFI ≤ 0.01 and ΔRMSEA ≤ 0.015. The reliability of the scales was estimated using *α* and *ω* and interpreted according to Watkins (2017).

Regarding the convergent validity, correlation coefficients were run with SPSS.27. Note that the missing values were not replaced in the study of the relationship between the three scales and the related variables (i.e., CC mitigation behaviors, self-efficacy toward CC, nature connectedness, and CC concern). Finally, two MANOVAs were run to compare boys and girls and school levels in the three modes of agency. The effect size of the variance analyses was assessed by the *χ*^2^ (small effect: *η*^2^ = 0.01; medium effect: *η*^2^ = 0.059; large effect: *η*^2^ = 0.138).

## 3 Results

### 3.1 Structural validity

Tables 1–3 show correlation coefficient data between the items of each agency mode. Table 4 presents the fit results of the factor models of the three agency modes (individual, proxy, and collective), and Table 5 presents the Standardized Regression Weights. Data show that the unifactor model has a limited and poorer fit than the other two models. All statistics show that models 2 and 3 present a better fit, including the AIC and BIC scores (individual agency: ΔAIC = 180.36, *p* < 0.001; ΔBIC = 165.31, *p* < 0.001; proxy agency: ΔAIC = 224.68, *p* < 0.001; ΔBIC = 209.63, *p* < 0.001; collective agency: ΔAIC = 677.30, *p* < 0.001; ΔBIC = 662.25, *p* < 0.001), which show an optimal and similar fit for the three agency modes. Therefore, models 2 and 3 are equally suitable to represent the factor structure of the scales for each agency mode.

**Table 1:** Pearson’s correlations between items of the individual agency mode [95% CI].

Individual agency items												
	I1	I2	I3	I4	I5	I6	I7	I8	I9	I10	I11	I12
I1	–											
I2	.496*** [450-.539]	–										
I3	.432*** [383-.479]	.478*** [.432-.522]	–									
I4	.558*** [.516-.597]	.564*** [.522-.603]	.575*** [.535-.613]	–								
I5	.411*** [.361-.459]	.477*** [.431-.521]	.481*** [.435-.525]	.548*** [.506-.588]	–							
I6	.477*** [.431-.521]	.552*** [.510-.591]	.477*** [.430-.521]	.594*** [.554-.630]	.586*** [.546-.623]	–						
I7	.362*** [.309-.412]	.461*** [.413-506]	.505*** [.460-.548]	.519*** [.474-.560]	.543*** [.500-.583]	.566*** [.525-.605]	–					
I8	.483*** [.436-.526]	.498*** [.452-.541]	.520*** [.476-.561]	.609*** [.570-.644]	.529*** [.485-.570]	.646*** [.611-.679]	.580*** [.540-.618]	–				
I9	.461*** [.414-.506]	.515*** [.470-.557]	.560*** [.518-.599]	.599*** [.560-.636]	.527*** [.484-.569]	.635*** [.598-.669]	.604*** [.565-.640]	.691*** [.659-.721]	–			
I10	.451*** [.403-.497]	.505*** [.460-.548]	.492*** [.447-.536]	.579*** [.538-.617]	.473*** [.426-.517]	.618*** [.581-.653]	.551*** [.509-.591]	.708*** [.677-.736]	.701*** [.670-.730]	–		
I11	.466*** [.418-.510]	.518*** [.473-560]	.504*** [.459-.547]	.555*** [.513-.595]	.501*** [.455-.543]	.615*** [.577-.650]	.545*** [.503-.585]	.636*** [.600-.670]	.649*** [.613-.681]	.675*** [.642-.706]	–	
I12	.412*** [.362-.459]	.496*** [.450-539]	.526*** [.483-.568]	.594*** [.554-.631]	.528*** [.485-.569]	.601*** [.562-.637]	.581*** [.540-.618]	.660*** [.625-.692]	.699*** [.668-.728]	.669*** [.635-.700]	.678*** [.645-.708]	–

CI, Confidence Intervals; I, Item; *** *p* < 0.001.

**Table 2:** Pearson’s correlations between items of the proxy agency mode [95% CI].

**Proxy Agency Items**												
	I1	I2	I3	I4	I5	I6	I7	I8	I9	I10	I11	I12
I1	–											
I2	.594*** [.554-.630]	–										
I3	.509*** [.464-.551]	.530*** [.487-.571]	–									
I4	.514*** [.469-.556]	.503*** [.457-.545]	.552*** [.510-.592]	–								
I5	.559*** [.517-.598]	.546*** [.503-.585]	.511*** [.467-.553]	.526*** [.482-.567]	–							
I6	.557*** [.515-596]	.587*** [.547-.624]	.539*** [.496-.580]	.575*** [.535-.613]	.652*** [.617-.685]	–						
I7	.521*** [.477-.563]	.542*** [.499-.582]	.494*** [.448-.537]	.504*** [.459-.547]	.597*** [.558-.634]	.641*** [.605-.674]	–					
I8	.559*** [.517-.598]	.524*** [.480-.565]	.523*** [.479-.564]	.543*** [.500-.583]	.630*** [.594-.664]	.669*** [.635-.700]	.685*** [.652-.715]	–				
I9	.523*** [.479-.564]	.544*** [.501-.584]	.538*** [.495-.578]	.559*** [.517-.598]	.585*** [.545-.622]	.630*** [.593-.664]	.614*** [.576-.649]	.734*** [.706-.760]	–			
I10	.517*** [.472-.558]	.541*** [.498-.581]	.532*** [.489-.573]	.564*** [.522-.603]	.611*** [.573-.647]	.670*** [.636-.701]	.615*** [.577-.650]	.705*** [.674-.733]	.721*** [.692-.748]	–		
I11	.524*** [.480-.565]	.544*** [.501-.584]	.498*** [.452-.541]	.530*** [.486-.571]	.602*** [.563-.638]	.659*** [.625-.691]	.638*** [.602-.671]	.687*** [.654-.717]	.680*** [.647-.710]	.733*** [.705-.759]	–	
I12	.492*** [.446-.535]	.563*** [.521-.602]	.498*** [.452-.541]	.519*** [.475-.561]	.601*** [.562-.637]	.619*** [.582-.654]	.623*** [.586-.658]	.695*** [.664-.725]	.703*** [.672-.732]	.710*** [.680-.738]	.712*** [.682-.740]	–

CI, Confidence Intervals; I, Item; *** *p* < 0.001.

**Table 3:** Pearson’s correlations between items of the collective agency mode [95% CI].

**Colective Agency Items**												
	I1	I2	I3	I4	I5	I6	I7	I8	I9	I10	I11	I12
I1	–											
I2	.672*** [.638-.703]	–										
I3	.595*** [.556-.632]	.623*** [.586-.658]	–									
I4	.670*** [.637-.701]	.652*** [.617-.684]	.663*** [.629-.695]	–								
I5	.511*** [.466-.553]	.516*** [.472-.558]	.463*** [.415-.507]	.575*** [.534-.613]	–							
I6	.577*** [.536-.615]	.608*** [.570-.644]	.558*** [.516-.597]	.658*** [.623-.690]	.726*** [.697-.753]	–						
I7	.537*** [.494-.577]	.580*** [.539-.617]	.526*** [.482-.567]	.587*** [.547-.624]	.649*** [.614-.682]	.739*** [.711-.764]	–					
I8	.560*** [.518-.599]	.593*** [.554-.630]	.568*** [.527-.607]	.643*** [.607-.676]	.667*** [.633-.698]	.741*** [.714-.767]	.732*** [.704-.758]	–				
I9	.556*** [.514-.595]	.549*** [.506-.588]	.572*** [.531-.610]	.637*** [.600-.670]	.536*** [.493-.577]	.622*** [.585-.657]	.646*** [.611-.679]	.731*** [.703-.757]	–			
I10	.539*** [.496-.580]	.564*** [.523-.603]	.544*** [.502-.584]	.623*** [.586-.658]	.603*** [.565-.639]	.700*** [.669-.729]	.668*** [.634-.699]	.763*** [.737-.787]	.765*** [.740-.788]	–		
I11	.549*** [.506-.588]	.568*** [.527-.607]	.530*** [.486-.571]	.625*** [.588-.660]	.662*** [.628-.694]	.722*** [.693-.749]	.706*** [.676-.735]	.762*** [.736-.786]	.707*** [.676-.735]	.775*** [.750-.797]	–	
I12	.570*** [.529-.609]	.575*** [.535-.613]	.548*** [.505-.588]	.635*** [.599-.669]	.617*** [.579-.652]	.705*** [.674-.734]	.708*** [.678-.737]	.747*** [.720-.772]	.728*** [.699-.754]	.785*** [.761-.806]	.791*** [.786-.812]	–

CI, Confidence Intervals; I, Item; *** *p* < 0.001.

**Table 4:** Model fit data of the three CFA models.

	*χ* ^2^	df	AGFI	TLI	CFI	RMR	RMSEA	AIC	BIC
Individual agency									
M1: Unifactorial	405.48	54	0.906	0.948	0.958	0.035	0.076	453.48	573.86
M2: Three-factors	219.12	51	0.949	0.974	0.980	0.026	0.054	273.12	408.55
M3: Multif./multilevel	219.12	51	0.949	0.974	0.980	0.026	0.054	273.12	408.55
Proxy agency									
M1: Unifactorial	434.09	54	0.898	0.950	0.959	0.036	0.080	482.09	602.47
M2: Three-factors	203.41	51	0.955	0.979	0.984	0.022	0.052	257.41	392.84
M3: Multif./multilevel	203.41	51	0.955	0.979	0.984	0.022	0.052	257.41	392.84
Collective agency									
M1: Unifactorial	978.44	54	0.772	0.901	0.919	0.064	0.124	1026.44	1146.82
M2: Three-factors	295.14	51	0.931	0.972	0.979	0.030	0.066	349.14	484.57
M3: Multif./multilevel	295.14	51	0.931	0.972	0.979	0.030	0.066	349.14	484.57

M1: Unifactorial (Figure 1 – Model 1a–c), M2: three factors (multifactors) (Figure 1 – Model 2a–c), M3: multifactorial/multilevel (Figure 1 – Model 3a–c).

**Table 5:** Standardized regression weights.

	Estimate	S.E.	C.R.	*p*
Individual agency				
Agency → Forethought	0.908	–	–	–
Agency → Self-Reactiveness	0.956	0.078	21.297	<0.001
Agency → Self-Reflectiveness	0.999	0.070	18.760	<0.001
Forethought → Item 1	0.653	–	–	–
Forethought → Item 2	0.706	0.059	20.154	<0.001
Forethought → Item 3	0.701	0.057	20.039	<0.001
Forethought → Item 4	0.820	0.058	22.606	<0.001
Self-Reactiveness → Item 5	0.683	–	–	–
Self-Reactiveness → Item 6	0.791	0.047	24.140	<0.001
Self-Reactiveness → Item 7	0.720	0.049	22.156	<0.001
Self-Reactiveness → Item 8	0.826	0.050	25.075	<0.001
Self-Reflectiveness → Item 9	0.843	–	–	–
Self-Reflectiveness → Item 10	0.826	0.028	33.756	<0.001
Self-Reflectiveness → Item 11	0.801	0.027	32.161	<0.001
Self-Reflectiveness → Item 12	0.824	0.028	33.612	<0.001
Proxy agency				
Agency → Forethought	0.908	–	–	–
Agency → Self-Reactiveness	0.949	0.057	24.322	<0.001
Agency → Self-Reflectiveness	0.999	0.055	23.077	<0.001
Forethought → Item 1	0.737	–	–	–
Forethought → Item 2	0.748	0.046	24.032	<0.001
Forethought → Item 3	0.713	0.048	22.868	<0.001
Forethought → Item 4	0.723	0.047	23.211	<0.001
Self-Reactiveness → Item 5	0.766	–	–	–
Self-Reactiveness → Item 6	0.815	0.035	29.068	<0.001
Self-Reactiveness → Item 7	0.783	0.039	27.67	<0.001
Self-Reactiveness → Item 8	0.847	0.037	30.462	<0.001
Self-Reflectiveness → Item 9	0.837	–	–	–
Self-Reflectiveness → Item 10	0.858	0.028	35.763	<0.001
Self-Reflectiveness → Item 11	0.842	0.028	34.667	<0.001
Self-Reflectiveness → Item 12	0.835	0.029	34.202	<0.001
Collective agency				
Agency → Forethought	0.859	–	–	–
Agency → Self-Reactiveness	0.955	0.052	25.255	<0.001
Agency → Self-Reflectiveness	0.983	0.048	24.078	<0.001
Forethought → Item 1	0.792	–	–	–
Forethought → Item 2	0.802	0.035	29.215	<0.001
Forethought → Item 3	0.767	0.039	27.232	<0.001
Forethought → Item 4	0.849	0.035	30.856	<0.001
Self-Reactiveness → Item 5	0.774	–	–	–
Self-Reactiveness → Item 6	0.866	0.033	32.242	<0.001
Self-Reactiveness → Item 7	0.837	0.035	30.577	<0.001
Self-Reactiveness → Item 8	0.883	0.037	32.502	<0.001
Self-Reflectiveness → Item 9	0.828	–	–	–
Self-Reflectiveness → Item 10	0.885	0.026	37.641	<0.001
Self-Reflectiveness → Item 11	0.884	0.026	37.025	<0.001
Self-Reflectiveness → Item 12	0.887	0.028	37.443	<0.001

### 3.2 Measurement invariance across sex and grade level

To study the invariance by sex and grade level, we used the two-factorial level model as the reference model because model 3 is more complete and presents a similar fit to model 2. The invariance by sex and grade level was examined for each agency mode (individual, proxy, and collective agency).

The sex invariance analysis of individual agency indicates that the fit of the base model is good [*χ*^2^(102) = 289.66, *p* < 0.05; TLI = 0.970; CFI = 0.977; RMR = 0.030; RMSEA = 0.041]. Acknowledging this finding, invariance was analyzed considering the (i) factorial weights (measurement weights) (ΔCFI = 0.000, ΔRMSEA = 0.002), (ii) structural weights (ΔCFI = 0.000, ΔRMSEA = 0.000), (iii) Structural model (structural covariances) (ΔCFI = 0.000, ΔRMSEA = 0.000), (iv) structural residuals (ΔCFI = 0.000, ΔRMSEA = 0.001), and (v) residuals of the measurements (measurement residuals) (ΔCFI = 0.006, ΔRMSEA = 0.002). Considering the sex invariance of proxy agency, data indicate that the base model fit is good [*χ*^2^(102) = 277.27, *p* < 0.05; TLI = 0.975; CFI = 0.981; RMR = 0.026; RMSEA = 0.039]. Invariance was analyzed considering the (i) factorial weights (measurement weights) (ΔCFI = 0.000, ΔRMSEA = 0.001), (ii) structural weights (ΔCFI = 0.000, ΔRMSEA = 0.001), (iii) Covariances of the structural model (structural covariances) (ΔCFI = 0.000, ΔRMSEA = 0.000), (iv) structural residuals (ΔCFI = 0.001, ΔRMSEA = 0.001), and (v) residuals of the measurements (measurement residuals) (ΔCFI = 0.001, ΔRMSEA = 0.001). Finally, we analyzed the sex invariance of collective agency. Data indicate that the fit of the base model is good [*χ*^2^(102) = 379.99, *p* < 0.05; TLI = 0.968; CFI = 0.976; RMR = 0.033; RMSEA = 0.050]. Consistently, invariance was studied while considering the (i) factorial weights (measurement weights) (ΔCFI = 0.001, ΔRMSEA = 0.002), (ii) structural weights (ΔCFI = 0.000, ΔRMSEA = 0.001), (iii) Covariances of the structural model (structural covariances) (ΔCFI = 0.000, ΔRMSEA = 0.000), (iv) structural residuals (ΔCFI = 0.001, ΔRMSEA = 0.000), and (v) measurements (measurement residuals) (ΔCFI = 0.001, ΔRMSEA = 0.001). Current results indicate that considering the five dimensions assessed (i.e., measurement weights, structural weights, covariances of the structural model, structural residuals, and measurement residuals) and the three agency modes (i.e., individual, proxy, and collective) data are invariant across sex.

We analyzed the grade level invariance of individual agency; data indicate that the fit of the base model is good [*χ*^2^(102) = 298.19, *p* < 0.05; TLI = 0.970; CFI = 0.977; RMR = 0.030; RMSEA = 0.042]. Grounded on this finding, invariance was analyzed considering the (i) factor weights (measurement weights) (ΔCFI = 0.001, ΔRMSEA = 0.002), (ii) structural weights – that is, the relationship between the factor general (mode) and the three first-order factors (proprieties) – (ΔCFI = 0.000, ΔRMSEA = 0.000), the (iii) Covariances of the structural model (ΔCFI = 0.000, ΔRMSEA = 0.001), (iv) structural residuals (ΔCFI = 0.000, ΔRMSEA = 0.001), and the (v) residuals of the measurements (measurement residuals) (ΔCFI = 0.002, ΔRMSEA = 0.001). Regarding the grade level invariance of proxy agency, data also indicate that the fit of the base model is good [*χ*^2^(102) = 256.63, *p* < 0.05; TLI = 0.979; CFI = 0.983; RMR = 0.024; RMSEA = 0.037]. Therefore, we analyzed invariance regarding the (i) factorial weights (measurement weights) (ΔCFI = 0.001, ΔRMSEA = 0.002), (ii) structural weights (ΔCFI = 0.000, ΔRMSEA = 0.000), (iii) Covariances of the structural model (structural covariances) (ΔCFI = 0.000, ΔRMSEA = 0.000), (iv) structural residuals (ΔCFI = 0.000, ΔRMSEA = 0.001), and (v) residuals of the measurements (measurement residuals) (ΔCFI = 0.006, ΔRMSEA = 0.004). Finally, considering the grade level invariance of collective agency, data indicate that the base model fit is good [*χ*^2^(102) = 372.10, *p* < 0.05; TLI = 0.969; CFI = 0.976; RMR = 0.034; RMSEA = 0.049]. Regarding the individual and proxy agency modes, invariance was analyzed considering the (i) factorial weights (measurement weights) (ΔCFI = 0.000, ΔRMSEA = 0.002), (ii) structural weights (ΔCFI = 0.000, ΔRMSEA = 0.000), (iii) Covariances of the structural model (structural covariances) (ΔCFI = 0.000, ΔRMSEA = 0.000), (iv) structural residuals (ΔCFI = 0.000, ΔRMSEA = 0.001), and (v) residuals of the measurements (measurement residuals) (ΔCFI = 0.008, ΔRMSEA = 0.004).

Current results indicate that data are invariant across grade levels when considering the five dimensions assessed (i.e., measurement weights, structural weights, covariances of the structural model, structural residuals, and measurement residuals) and the three agency modes (i.e., individual, proxy, and collective).

### 3.3 Reliability

The reliability of the three agency modes is very good: individual agency (αT1 = 0.94; ωT1 = 0.95; αT2 = 0.95; ωT2 = 0.95); proxy agency (αT1 = 0.94; ωT1 = 0.94; αT2 = 0.96; ωT2 = 0.96) and collective agency (αT1 = 0.95; ωT1 = 0.95; αT2 = 0.97; ωT2 = 0.97). In addition, Pearson’s correlation coefficients between T1 and T2 for individual (0.768), proxy (0.738), and collective (0.767) agency modes are high.

### 3.4 Convergent validity

As Table 6 shows, the bold correlations for the three agency modes indicate convergent validity. Specifically, the three modes of agency are positively and significantly correlated with the four related variables (i.e., individual mitigation behaviors, self-efficacy, nature connectedness, and CC concern). Data indicate that the higher the agency modes scores (individual, proxy, and collective), the higher the individual mitigation behaviors, self-efficacy, nature connectedness, and CC concern, and vice versa.

**Table 6:** Pearson correlation coefficients [95% CI].

	IA	PA	CA	MB	SE	NC	CCC
IA	–						
PA	0.803** [0.781–0.823]	–					
CA	0.636** [0.599–0.669]	0.740** [0.713–0.766]	–				
MB	0.425** [0.376–0.472]	0.342** [0.289–0.393]	0.336** [0.283–0.387]	–			
SE	0.631** [0.577–0.679]	0.515** [0.451–0.575]	0.462** [0.393–0.526]	0.442** [0.372–0.507]	–		
NC	0.380** [0.307–0.448]	0.411** [0.340–0.477]	0.345** [0.270–0.416]	0.283** [0.205–0.357]	x	–	
CCC	0.532** [0.470–0.589]	0.449** [0.380–0.513]	0.344** [0.269–0.415]	0.315** [0.238–0.388]	x	0.277** [0.199–0.352]	–

CI (Confidence Intervals), IA (individual agency), PA (proxy agency), CA (collective agency), MB (mitigation behaviors), SE (self-efficacy), NC (nature connectedness), CCC (climate change concern). **p < 0.01. x = Variables assessed in different versions of the questionnaire (SE in version A, and NC and CCC in version B). The number of participants who fulfilled each scale varied: agency modes (*N* = 1,114), mitigation behaviors (*N* = 1,114), self-efficacy (*n* = 545), nature connectedness (*n* = 566; 3 missing values), and climate change concern (*n* = 558; 11 missing values). In the relationship between IA, PA, and CA with the external variables (i.e., mitigation behaviors, self-efficacy, nature connectedness, and climate change concern), the missing values for these variables have not been replaced. Correlations of interest are in bold.

### 3.5 Sex and grade level differences in individual, proxy, and collective agency

The differences in the three modes of agency regarding sex and grade level were analyzed. Table 7 shows data for the means and standard deviations. Regarding the variable sex, we also found statistically significant differences [*λ*_wilks_ = 0.997; *F*(3,1,109) = 8.790; *p* < 0.001; *η*^2^ = 0.023]. Girls scored higher than boys in the three agency modes: individual [*F*(1,111) = 25.26; *p* < 0.001; *η*^2^ = 0.022], proxy [*F*(1,111) = 16.09; *p* < 0.001; *η*^2^ = 0.014] and collective [*F*(1,111) = 15.08; *p* < 0.001; *η*^2^ = 0.013]. Regarding the variable grade level, we found statistically significant differences [*λ*_wilks_ = 0.957; *F*(15,3,053) = 3.283; *p* < 0.001; *η*^2^ = 0.015] there is generally a tendency showing that the agency modes decrease as the grade level grows: individual [*F*(5,1,108) = 2.97; *p* < 0.05; *η*^2^ = 0.013], proxy [*F*(5,1,108) = 4.68; *p* < 0.001; *η*^2^ = 0.021] and collective [*F*(5,1,108) = 4.41; *p* < 0.001; *η*^2^ = 0.020]. However, considering sex and grade level, the effect sizes of the differences found are small.

**Table 7:** Means and standard deviations.

		Sex		Grade Level					
		Girl	Boy	7^th^	8^th^	9^th^	10^th^	11^th^	12^th^
Individual Agency									
	*M*	3.318	3.078	3.294	3.079	3.307	3.163	3.160	3.095
	*SD*	0.769	0.819	0.829	0.784	0.770	0.816	0.750	0.899
Proxy Agency									
	*M*	2.771	2.573	2.828	2.613	2.753	2.675	2.526	2.480
	*SD*	0.813	0.829	0.848	0.783	0.799	0.882	0.769	0.904
Collective Agency									
	*M*	2.523	2.307	2.626	2.404	2.410	2.387	2.290	2.187
	*SD*	0.911	0.945	0.985	0.883	0.924	0.954	0.878	0.9055

M (Mean), SD (Standard Deviation).

## 4 Discussion

The current work aimed to fill a three-fold research gap concerning the absence of quantitative measures to assess adolescents’ agency modes toward CC. Hence, the current work followed two phases: (i) developing three theory-based scales to measure each agency mode toward CC (i.e., individual, proxy, and collective), and (ii) testing and validating those scales.

Regarding the first phase, items were generated based on the description of the human agency construct and its properties, according to Bandura’s (2018) theory. This procedure was essential to fully capture the different components of the construct (Tsang et al., 2017). The evaluation of the scales by five expert judges helped support their content validity (i.e., items measure what is supposed to be measured), constituting a prerequisite to assure other types of instrument validity (Yusoff, 2019). As crucial as this procedure was the evaluation by the target population to ensure that participants understood the items of the scales (Arafat et al., 2016). Altogether, these methodological procedures provided us with preliminary evidence that the items of the developed scales assess agency modes toward CC and their properties (Boateng et al., 2018; Tsang et al., 2017).

In the second phase, we examined the psychometric properties of the scales mentioned above. According to Bandura (2018), each agency mode comprises three properties: forethought, self-reactiveness, and self-reflectiveness. CFA supported the theoretical structure of the scales for each agency mode. Specifically, Model 2 (i.e., first-order multifactorial structure of each agency mode) and Model 3 (i.e., one second-order factor and first-order multifactorial structure of each agency mode) have an identical fit. Hence, we can use both, i.e., the mean scores of each property and the mean score of each agency mode. Moreover, tests for group invariance indicated that this model is consistent across sex (H2) and grade levels (H3), which allows the comparison between those groups. This means that differences in the scale scores reflect actual group differences in agency toward CC rather than potential measurement bias, which strengthens the validity of each agency mode scale (Cheung and Rensvold, 2002).

In addition, each agency mode scale exhibited excellent psychometric properties in terms of internal reliability at T1 and T2 (H4–H6), reflecting adequate homogeneity (Nunnally and Bernstein, 1994). Correlation coefficients between T1 and T2 for individual (0.768), proxy (0.738), and collective (0.767) agency modes are high, indicating the stability of participants’ responses over time (H7–H9).

Concerning convergent validity, we confirmed all hypotheses. Based on literature focused on related variables, i.e., pro-environmental or CC mitigation behaviors (e.g., Busch et al., 2019; Krettenauer et al., 2020; Lawson et al., 2019), we expected positive and statistically significant relationships between all agency modes and CC mitigation behaviors (H10), self-efficacy to combat CC (H11), nature connectedness (H12), and CC concern (H13). As Table 6 shows, correlation coefficients are stronger between agency modes and self-efficacy, nature connectedness, and CC concern (i.e., correlation coefficients range between 0.344 and 0.631) than with CC mitigation behaviors (correlation coefficients range between 0.283 and 0.442). Those results stress the need to investigate further the relationships between individual and contextual predictors of adolescents’ agency modes toward CC.

Lastly, we found that girls report being more agentic toward CC in all modes than boys (H14), which is consistent with the literature on learning agency and self-regulation (e.g., Martins et al., 2024; Motie et al., 2012; Zimmerman and Martinez-Pons, 1990), as well as on pro-environmental or CC mitigation behaviors (Fielding and Head, 2012; Zeeshan et al., 2021). Moreover, we also found that older students report lower agency toward CC (all agency modes) than younger students (H15), consistent with the literature on pro-environmental or CC mitigation behaviors (e.g., Krettenauer, 2017; Negev et al., 2008). This data shows that despite greater cognitive development and autonomy that can be favorable to CC cause (e.g., Byrnes, 2006; Ojala and Lakew, 2017; Zimmer-Gembeck and Collins, 2006), high school students reported lower agency modes than their counterparts. This result highlights the need to intervene with students during high school level to counter the decreasing tendency to act toward CC and mitigate the known “adolescence dip” (e.g., Olsson and Gericke, 2016; Otto et al., 2019).

In sum, results showed that AGENTC2-Scales have good psychometric quality regarding validity and reliability evidence. Hence, these scales are of good value for assessing and identifying potential differences in the reported agency modes, as well as in the reported agency properties within each agency mode. This information may help educators establish educational goals to promote each property of the three agency modes. Only promoting forethought (e.g., designing a plan to combat CC), self-reactiveness (e.g., changing the strategies to combat CC), and self-reflectiveness (e.g., reflecting on the effectiveness of actions to combat CC) competencies together will contribute to developing real agents toward the desired outcome, i.e., mitigate CC (Bandura, 2018). In other words, combating CC requires more than simply mitigation actions; students need to develop anticipatory and reflective thinking to initiate, sustain, and improve those actions (Koskela and Paloniemi, 2023). Moreover, the scales can be used to assess the impact of CC formal education and intervention programs on students’ agency modes (and properties) toward CC. Notwithstanding the scales’ strengths and their contribution to research, this study has some limitations. Despite a good sample size, the number of participants at middle and high school levels is not balanced. Besides, despite our efforts to recruit participants from all regions of the country, most schools are from the North. Future studies could overcome this limitation. Lastly, future research could also extend the validation of the agency modes scales in other countries and the adult population.

## Data Availability

The raw data supporting the conclusions of this article will be made available by the authors, without undue reservation.
