# Supplementary material for: Adolescents’ agency toward climate change: development and validation of scales for individual, proxy, and collective modes
Source: Front Psychol. 2025 May 5;16:1532409. doi: 10.3389/fpsyg.2025.1532409 (PMC12089972; doi:10.3389/fpsyg.2025.1532409)
Supplement: Supplementary file 1 [file Supplementary_file_1.docx]

Supplementary Material

Adolescents’ Agency Toward Climate Change: Development and Validation of Scales for Individual, Proxy, and Collective Modes

Jennifer Cunha^1*^, Juliana Martins^1^, José Carlos Núñez^2^, Guillermo Vallejo^2^, Pedro Rosário^1^

^1^Psychology Research Center, School of Psychology, University of Minho, Braga, Portugal

^2^Departamento de Psicología, Universidad de Oviedo, Oviedo, Spain

*** Correspondence:**Corresponding Author
jcunha@psi.uminho.pt

Supplementary Tables and Figure are provided in the following pages.

# Supplementary Tables

**Table 1.** Items of Each Agency Mode Scale, Organized by Core Properties.

| **Core properties**  **Definition** | **Individual Agency Mode** | **Proxy Agency Mode** | **Collective Agency Mode** |
| --- | --- | --- | --- |
| (A) Forethought involves “the temporal extension of agency” and “set[ting] goals and anticipat[ing] likely outcomes of prospective actions to guide and motivate their efforts” (Bandura, 2006, p. 164). | A1. I imagine possible situations (e.g., at home or school) where I can combat climate change.  A2. I think about the possible consequences of my behaviors when I set a plan to reduce climate change.  A3. I think about the future of the planet in the coming years if I don’t do anything to combat climate change.  A4. I think about the different actions I can take to combat climate change. | A1. I imagine possible situations where I can influence people with knowledge and/or resources to combat climate change when it is not within my reach to do it alone.  A2. I think about the possible consequences of my behaviors when I set a plan to influence people with knowledge and/or resources to combat climate change.  A3. I think about the future of the planet in the coming years if I don’t influence people with knowledge and/or resources to combat climate change.  A4. I think about the different actions (e.g., informal conversations, social media posts) that can contribute to influencing people with knowledge and/or resources to combat climate change when it is not within my reach to do it alone. | A1. I imagine possible situations where I can combat climate change with groups/associations/institutions through collective actions.  A2. I think about the possible consequences of my behaviors when I set a plan to combat climate change with groups/associations/institutions.  A3. I think about the future of the planet in the coming years if I don’t carry out actions with groups/associations/institutions to combat climate change.  A4. I think about the different actions I can take with groups/associations/institutions to combat climate change. |
| (B) Self-reactiveness is to motivate and regulate the execution of action plans” (Bandura, 2001, p. 8). | B1. I carry out environmentally friendly actions to fulfill my plan to reduce climate change.  B2. I keep analyzing my strategies and actions to achieve my goal of reducing climate change.  B3. I change my behaviors when I realize they do not help combat climate change.  B4. I try to stay motivated to combat climate change. | B1. I influence people with knowledge and/or resources to combat climate change when it is not within my reach to do it alone.  B2. I keep analyzing my strategies and actions to influence people with knowledge and/or resources to help me achieve my goal of reducing climate change.  B3. I change my strategies when I realize I am not able to influence people with knowledge and/or resources to combat climate change.  B4. I try to stay motivated to influence people with knowledge and/or resources to combat climate change when it is not within my reach to do it alone. | B1. I carry out actions with groups/associations/institutions to combat climate change.  B2. I keep analyzing the strategies and actions I carry out with groups/associations/institutions to achieve my goal of reducing climate change.  B3. I change my strategies when I realize I am not able to carry out actions with groups/associations/institutions to combat climate change.  B4. I try to stay motivated to combat climate change with groups/associations/institutions. |
| (C)  Self-reflectiveness involves reflection on “their personal efficacy, the soundness of their thoughts and actions, and the meaning of their pursuits and they make corrective adjustments if necessary” (Bandura, 2006a, p. 165). | C1. I think about the importance of my goal of reducing climate change.  C2. I reflect on my motivation to combat climate change.  C3. I reflect on whether I managed to adopt effective behaviors to combat climate change and conclude whether it went as planned.  C4. I think about what I have done and how I can improve to combat climate change. | C1. I think about the importance of influencing people with knowledge and/or resources to combat climate change when it is not within my reach to do it alone.  C2. I reflect on my motivation to influence other people with knowledge and/or resources to combat climate change when it is not within my reach to do it alone.  C3. I reflect on what I did to influence people with knowledge and/or resources to combat climate change and conclude whether it went as planned.  C4. I think about what I have done and how I can improve to influence people with knowledge and/or resources to combat climate change. | C1. I think about the importance of participating in actions with groups/associations/institutions to combat climate change.  C2. I reflect on my motivation to combat climate change with groups/associations/institutions.  C3. I reflect on my participation in actions with groups/associations/institutions to combat climate change and conclude whether it went as planned.  C4. I think about what I have done and how I can improve to combat climate change with groups/associations/institutions. |

*Note*. Items were alternated in the version administrated in the study (see Appendix B). A bilingual researcher translated the Portuguese version of the items into English. Response options range from 1 to 5 as follows: 1. Never; 2. A few times; 3. Sometimes; 4. A lot of times; 5. Always.

**Table 2.** Adolescents’ Agency Toward Climate Change Scales (AGENTC^2^-S) – Translated Version Into English

| **To combat climate change you can act in different ways, for example, through:** | | |
| --- | --- | --- |
| **Individual action** - things you can do yourself daily (e.g., recycling, saving energy). | **Influential action** - influencing other people with knowledge and/or resources to do something when you can’t do it by yourself (e.g., influencing your family members to buy an electric appliance with more efficient energy consumption or speaking with a local politician to implement measures to fight climate change). | **Group action** - when you join groups (e.g., class, youth groups, clubs, scouts) or associations/institutions and work as a team (e.g., collecting trash/garbage on beaches, participating in protests, or organizing debates at school). |
| **Next, you will have to indicate how often you think of and perform each action.** Right or wrong responses do not exist. The important thing is that you feel free to choose the option that best fits your case.  1. Never 2. A few times 3. Sometimes 4. A lot of times 5. Always | | |
| A1. I imagine possible situations (e.g., at home or school) where I can combat climate change. | A1. I imagine possible situations where I can influence people with knowledge and/or resources to combat climate change when it is not within my reach to do it alone. | A1. I imagine possible situations where I can combat climate change with groups/associations/institutions through collective actions. |
| B1. I carry out environmentally friendly actions to fulfill my plan to reduce climate change. | B1. I influence people with knowledge and/or resources to combat climate change when it is not within my reach to do it alone. | B1. I carry out actions with groups/associations/institutions to combat climate change. |
| C1. I think about the importance of my goal of reducing climate change. | C1. I think about the importance of influencing people with knowledge and/or resources to combat climate change when it is not within my reach to do it alone. | C1. I think about the importance of participating in actions with groups/associations/institutions to combat climate change. |
| A2. I think about the possible consequences of my behaviors when I set a plan to reduce climate change. | A2. I think about the possible consequences of my behaviors when I set a plan to influence people with knowledge and/or resources to combat climate change. | A2. I think about the possible consequences of my behaviors when I set a plan to combat climate change with groups/associations/institutions. |
| B2. I keep analyzing my strategies and actions to achieve my goal of reducing climate change. | B2. I keep analyzing my strategies and actions to influence people with knowledge and/or resources to help me achieve my goal of reducing climate change. | B2. I keep analyzing the strategies and actions I carry out with groups/associations/institutions to achieve my goal of reducing climate change. |
| C2. I reflect on my motivation to combat climate change. | C2. I reflect on my motivation to influence other people with knowledge and/or resources to combat climate change when it is not within my reach to do it alone. | C2. I reflect on my motivation to combat climate change with groups/associations/institutions. |
| A3. I think about the future of the planet in the coming years if I don’t do anything to combat climate change. | A3. I think about the future of the planet in the coming years if I don’t influence people with knowledge and/or resources to combat climate change. | A3. I think about the future of the planet in the coming years if I don’t carry out actions with groups/associations/institutions to combat climate change. |
| B3. I change my behaviors when I realize they do not help combat climate change. | B3. I change my strategies when I realize I am not able to influence people with the knowledge and/or resources to combat climate change. | B3. I change my strategies when I realize I am not able to carry out actions with groups/associations/institutions to combat climate change. |
| C3. I reflect on whether I managed to adopt effective behaviors to combat climate change and conclude whether it went as planned. | C3. I reflect on what I did to influence people with knowledge and/or resources to combat climate change and conclude whether it went as planned. | C3. I reflect on my participation in actions with groups/associations/institutions to combat climate change and conclude whether it went as planned. |
| A4. I think about the different actions I can take to combat climate change. | A4. I think about the different actions (e.g., informal conversations, social media posts) that can contribute to influencing people with knowledge and/or resources to combat climate change when it is not within my reach to do it alone. | A4. I think about the different actions I can take with groups/associations/institutions to combat climate change. |
| B4. I try to stay motivated to combat climate change. | B4. I try to stay motivated to influence people with knowledge and/or resources to combat climate change when it is not within my reach to do it alone. | B4. I try to stay motivated to combat climate change with groups/associations/institutions. |
| C4. I think about what I have done and how I can improve to combat climate change. | C4. I think about what I have done and how I can improve to influence people with knowledge and/or resources to combat climate change. | C4. I think about what I have done and how I can improve to combat climate change with groups/associations/institutions. |

*Note*. Letters represent each core property: A = Forethought; B = Self-reactiveness; C = Self-reflectiveness. A bilingual researcher translated the Portuguese version of the items into English.

**Table 3.** Adolescents’ Agency Toward Climate Change Scales (AGENTC^2^-S) – Portuguese Version

| **Para combater as alterações climáticas podes agir de diferentes formas, por exemplo, através da:** | | |
| --- | --- | --- |
| **Ação individual** - coisas que consegues fazer sozinho(a) no teu dia-a-dia (ex., reciclar, poupar energia). | **Ação de influência** - influenciar outras pessoas com conhecimentos e/ou recursos a fazer algo quando não consegues fazer sozinho(a) (ex., influenciar os teus familiares a comprarem um eletrodoméstico com consumo energético mais eficiente ou falar com o Presidente da Junta para implementar medidas para combater as alterações climáticas). | **Ação em grupo** - quando te juntas a grupos (ex., turma, grupos de jovens, clubes, escuteiros) ou associações/instituições e trabalham em equipa (ex., recolha de lixo nas praias, participar em manifestações, organizar debates na minha escola). |
| **De seguida, terás de indicar a frequência com que pensas e/ou realizas cada uma destas ações.** Não existem respostas certas nem erradas. O importante é que te sintas à vontade para escolher a opção com que mais te identificas/que melhor se ajusta ao teu caso.  1. Nunca 2. Poucas vezes 3. Algumas vezes 4. Muitas vezes 5. Sempre | | |
| A1. Imagino possíveis situações (ex., em casa ou na escola) em que poderei combater as alterações climáticas. | A1. Imagino possíveis situações em que poderei influenciar pessoas com conhecimento e/ou recursos a combaterem as alterações climáticas quando não está ao meu alcance fazê-lo sozinho(a). | A1. Imagino possíveis situações em que poderei  combater as alterações climáticas com  grupos/associações/instituições através da realização de ações coletivas. |
| B1. Realizo ações amigas do ambiente para cumprir o meu plano de diminuir as alterações climáticas. | B1. Influencio pessoas com conhecimento e/ou recursos a combaterem as alterações climáticas quando não está ao meu alcance fazê-lo sozinho(a). | B1. Realizo ações com grupos/associações/instituições para combater as alterações climáticas. |
| C1. Penso na importância do meu objetivo de diminuir as alterações climáticas. | C1. Penso na importância de influenciar pessoas com conhecimento e/ou recursos a combaterem as  alterações climáticas quando não está ao meu alcance fazê-lo sozinho(a). | C1. Penso na importância de participar em ações com grupos/associações/instituições para combater as alterações climáticas. |
| A2. Penso em possíveis consequências dos meus comportamentos quando defino um plano para diminuir as alterações climáticas. | A2. Penso em possíveis consequências dos meus  comportamentos quando defino um plano para  influenciar pessoas com conhecimento e/ou recursos a combaterem as alterações climáticas. | A2. Penso em possíveis consequências dos meus  comportamentos quando defino um plano para  combater as alterações climáticas com grupos/associações/instituições. |
| B2. Vou analisando as minhas estratégias e ações, para atingir o meu objetivo de diminuir as alterações climáticas. | B2. Vou analisando as minhas estratégias e ações para influenciar pessoas com conhecimento e/ou recursos para me ajudarem a atingir o meu objetivo de diminuir as alterações climáticas. | B2. Vou analisando as estratégias e as ações que realizo com grupos/associações/instituições, de forma a atingir o meu objetivo de diminuir as alterações climáticas. |
| C2. Reflito sobre a minha motivação para combater as alterações climáticas. | C2. Reflito sobre a minha motivação para influenciar outras pessoas com conhecimento e/ou recursos a combaterem as alterações climáticas quando não está ao meu alcance fazê-lo sozinho(a). | C2. Reflito sobre a minha motivação para combater as alterações climáticas com grupos/associações/instituições. |
| A3. Penso como será o futuro do planeta nos próximos anos se não fizer nada para combater as alterações climáticas. | A3. Penso como será o futuro do planeta nos próximos anos se não influenciar pessoas com conhecimento e/ou recursos a combaterem as alterações climáticas. | A3. Penso como será o futuro do planeta nos próximos anos se não realizar ações com grupos/associações/instituições para combater as alterações climáticas. |
| B3. Altero os meus comportamentos quando percebo que não ajudam a combater as alterações climáticas. | B3. Altero as minhas estratégias quando percebo que não estou a conseguir influenciar pessoas com conhecimento e/ou recursos a combaterem as alterações climáticas. | B3. Altero as minhas estratégias quando percebo que não estou a conseguir realizar ações com  grupos/associações/instituições para combater as alterações climáticas. |
| C3. Reflito se consegui adotar comportamentos eficazes para combater as alterações climáticas e concluo se correu como planeado. | C3. Reflito sobre o que fiz para influenciar pessoas com conhecimento e/ou recursos a combaterem as alterações climáticas e concluo se correu como planeado. | C3. Reflito sobre a minha participação nas ações com grupos/associações/instituições para combater as alterações climáticas e concluo se correu como planeado. |
| A4. Penso em diferentes ações que eu posso fazer para combater as alterações climáticas. | A4. Penso em diferentes ações (ex., conversas  informais, publicações nas redes sociais) que podem contribuir para influenciar pessoas com conhecimento e/ou recursos a combaterem as alterações climáticas quando não estiver ao meu alcance fazê-lo sozinho(a). | A4. Penso em diferentes ações que posso fazer com  grupos/associações/instituições para combater as  alterações climáticas. |
| B4. Tento manter-me motivado(a) para combater as  alterações climáticas. | B4. Tento manter-me motivado(a) para influenciar pessoas com conhecimento e/ou recursos a combaterem as alterações climáticas quando não está ao meu alcance fazê-lo sozinho(a). | B4. Tento manter-me motivado(a) para combater as  alterações climáticas com grupos/associações/instituições. |
| C4. Penso no que fiz e como posso melhorar para  combater as alterações climáticas. | C4. Penso no que fiz e como posso melhorar para  influenciar pessoas com conhecimento e/ou recursos a combaterem as alterações climáticas. | C4. Penso no que fiz e como posso melhorar para  combater as alterações climáticas com grupos/associações/instituições. |

*Note*. Letters represent each core property: A = Forethought; B = Self-reactiveness; C = Self-reflectiveness. The Portuguese version of the scales was administered in the current study.

# Supplementary Figure

**
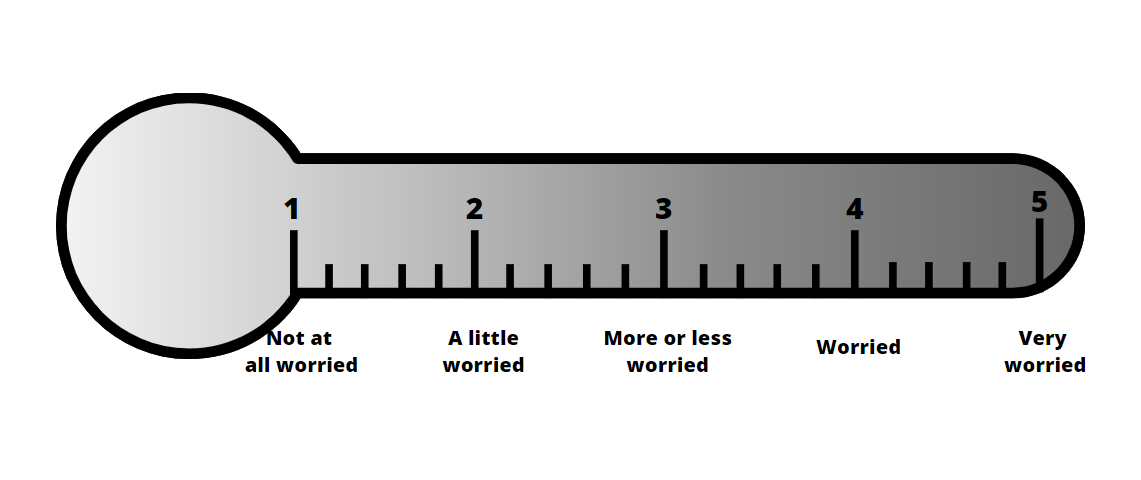
**

**Supplementary Figure 1.** Worry thermometer.
